# Soap Which Heals

**Published:** 1875-08

**Authors:** 


### SOAP WHICH HEALS.

Questions come from many readers concerning Packers' Tar Soap—to which we alluded in our last issue—and while we cannot answer all in detail, we are willing to give a condensed reply, which will meet most cases.

The fact that pure pine tar has a "healing" tendency is unquestioned. Every doctor will tell you that what are known as the terebinthinates, the balsams, are "healing" in their effect. Balsam of Fir, Tolu and Copaiba are used in irritations and ulcerations of the mucus surfaces, and their influence is specific upon such ailaments. They would be useful in skin diseases as well, if they could be conveniently applied. But the balsams, like tar, are sticky and not cleanly to smear upon the skin, and therefore they are not used. Mingled with soap, merely *stirred in*, as has been the practice in the manufacture of all tar-soaps, amounts to nothing, as no considerable proportion of tar or balsam can be thus incorporated. What is needed is to *saponify the tar*. This makes it soluble in water, and enables it to be brought in contact with the skin without offence.

Packer has found out how to make a soap from a basis of pure pine tar and cocoanut oil. Any one who uses it will be ready to testify that tar is its principal ingredient. It cleanses well, it affords a fine lather, and as a toilet soap its employment is really delightful. In hot weather, a daily bath is a necessity if one would keep sweet, and even then the odor of perspiration is apt to become quite offensive. To overcome this, stir your cake of tar soap around in your wash-bowl or bath-tub, and you will speedily discover that the bad smells are neutralized. If the scalp is tender or subject to pimples and dandruff or irritation, this soap of Packers has a wonderfully soothing and healing influence.

